# Pulsatile Tinnitus due to a Tortuous Siphon-Like Internal Carotid Artery Successfully Treated by Arterial Remodeling

**DOI:** 10.1155/2013/938787

**Published:** 2013-03-31

**Authors:** Dirk De Ridder, Sven Vanneste, Tomas Menovsky

**Affiliations:** TRI Tinnitus Clinic, BRAI2N & Department of Neurosurgery, Antwerp University Hospital, 2650 Edegem, Belgium

## Abstract

A patient is described with a right-sided tortuous siphon-like extracranial internal carotid artery leading to highly distressing ipsilateral heart beat synchronous pulsatile tinnitus, scoring 9/10 measuring loudness. Dilating the balloon during the occlusion test in or distal to the siphon-like anomaly reduces the arterial pulsations. Subsequently, surgery is performed using Teflon as an external construct to straighten the siphon-like anomaly. Postoperatively, the pulsations improve to 5/10 in a standing position and disappear during a reclined position. By adding a hearing aid, the pulsations are almost completely gone during a standing position (1/10) and remain absent in a reclined position.

## 1. Introduction

Tinnitus is a common symptom affecting 10%–15% of the population [1]. The most common form of tinnitus is nonpulsatile tinnitus, which is most often related to hearing loss, and therefore has been considered as an auditory phantom percept, analogous to phantom pain [2]. Pulsatile tinnitus is not as common as nonpulsatile tinnitus. It is most commonly seen in the presence of abnormal extracranial or intracranial blood vessels or intracranial hypertension, and it is not related to an abnormally functioning auditory system.

Pulsatile tinnitus can be subdivided into arterial heart beat synchronous or venous “hum-like” pulsatile tinnitus [3]. It can sometimes be heard by the clinician, in which case it is called objective tinnitus. The perceived pulsations are most likely transmitted via the cerebrospinal fluid to the cochlea [4], a mechanism similar to what has been proposed as an explanation for bone conduction [5–7]. 

Since heart beat synchronous tinnitus is predominantly vascular in origin, almost all causes of pulsatile tinnitus, except benign intracranial hypertension [8–10], can be diagnosed by different forms of angiography, such as classical intra-arterial or intravenous angiography, CT angiography, or more frequently magnetic resonance angiography. 

A common cause of arterial pulsatile tinnitus is carotid stenosis [8–10], often linked to atherosclerotic disease [11, 12], but a stenotic subclavian [13] or external carotid artery [14] or reversal of blood flow in an aberrant occipital artery can also cause pulsatile tinnitus [15]. Basically, any disease that changes the flow pattern into a turbulent flow can cause pulsatile tinnitus. Fibromuscular dysplasia is such an example [16, 17] as is a turbulent flow generated by looping of the AICA in the internal acoustic canal [4, 18]. Arterial heart beat synchronous pulsatile tinnitus associated with stenosis of the extra- or intracranial carotid artery typically disappears when compressing the ipsilateral carotid artery. The diagnosis can be confirmed by ultrasound sonography, MRI, CT, or classical angiography. Correction of the carotid artery stenosis can be achieved by dilation and stenting or carotid endarterectomy, and this abolishes the turbulent flow and the clinical signs associated with it. Indeed, ipsilateral carotid endarterectomy effectively reduces or abolishes the pulsatile tinnitus in more than 90% of patients with demonstrated ICA stenosis. Proximal lesions in the neck lend themselves to carotid endarterectomy, whereas distal lesions have been treated by stenting [19]. For the rarer intracranial carotid artery stenosis or stenosis of the carotid in the skull base, two approaches have been used; an initial balloon occlusion test under transcranial doppler and EEG monitoring can verify whether the ipsilateral carotid artery can be sacrificed. If so, one option is to ligate the symptomatic carotid artery and thereby arrest the turbulent flow. The second option is to dilate and stent the intracranial portion of carotid artery, resulting in a disappearance of the arterial pulsatile tinnitus [20]. Overall, almost 70% of patients with carotid stenosis are cured by intervention, and most of these patients experience (close to 90%) immediate relief of tinnitus [19].

In this paper, a patient is described with a tortuous extracranial internal carotid artery leading to ipsilateral pulsatile tinnitus successfully treated by surgical arterial remodeling. 

## 2. Case Description

This 74-year-old woman presented at the multidisciplinary TRI clinic (Tinnitus Research Initiative) of the Antwerp University Hospital, Belgium, with intractable unilateral right-sided pulsatile heart beat synchronous tinnitus since one year. The tinnitus scored *9/10* on a visual analogue scale for loudness. She had a TQ [21] score of 58, that is, a grade III, in other words, very severe without psychological decompensation. She had a high frequency hearing loss compatible with presbyacusis.

Previous unsuccessful treatments consisted of medication (flunarizine, gingko biloba, carbamazepine, sertraline, alprazolam, gabapentin, and topiramate), acupuncture, low level laser therapy, and transcranial magnetic stimulation, but without success.

### 2.1. Imaging Studies

**Figure 1:**
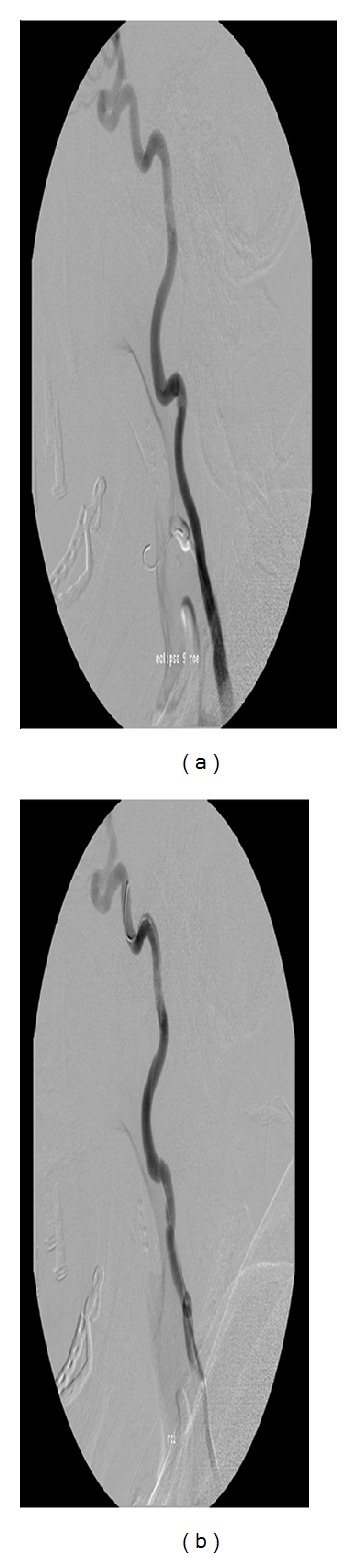
Loop in ICA (a) decreases on stretching by catheter in ICA (b), associated with a decrease in pulsatile tinnitus perception.

**Figure 2:**
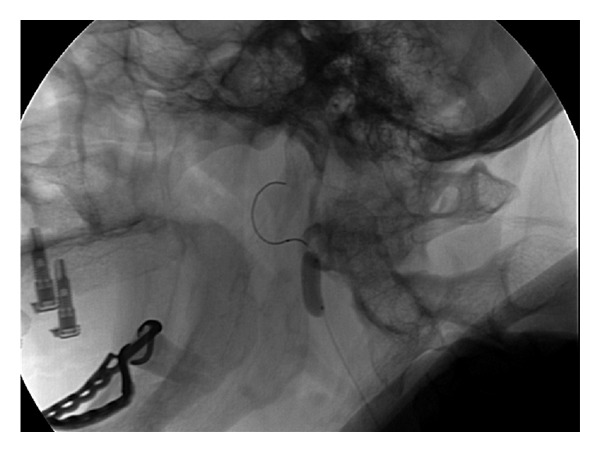
Balloon occlusion test reduced the pulsatile tinnitus almost completely.

MR imaging revealed no abnormalities and specifically no neurovascular conflict at the cerebellopontine angle. CT-angiography and a conventional angiography revealed a tortuous elongated internal carotid artery with a remarkable siphon-like loop just distal from the bifurcation of the common carotid artery (Figures 1 and 2). The patient was scheduled for an angiography and a balloon occlusion test to see whether she would benefit from occlusion of the external carotid artery.

### 2.2. Digital Subtraction Angiography and Balloon Occlusion

Cerebral angiography revealed indeed an arterial loop of the right extracranial internal carotid artery (Figure 1(a)). Occlusion of the external carotid artery did not improve the tinnitus; however, it was observed that temporary stretching of the right common carotid artery induced by the endovascular catheter during the angiography diminished the pulsatile tinnitus (Figure 1(b)). This observation raised the possibility that the kinking in the internal carotid artery was responsible for the tinnitus. 

The internal carotid artery was temporarily occluded by a balloon both proximally, in and distally of the siphon, always resulting in a remarkable improvement of the pulsatile tinnitus (Figure 2). As a consequence, the option of surgically remodeling the internal carotid artery was discussed with the patient and the patient gave an informed consent to the surgery.

### 2.3. Surgery

**Figure 3:**
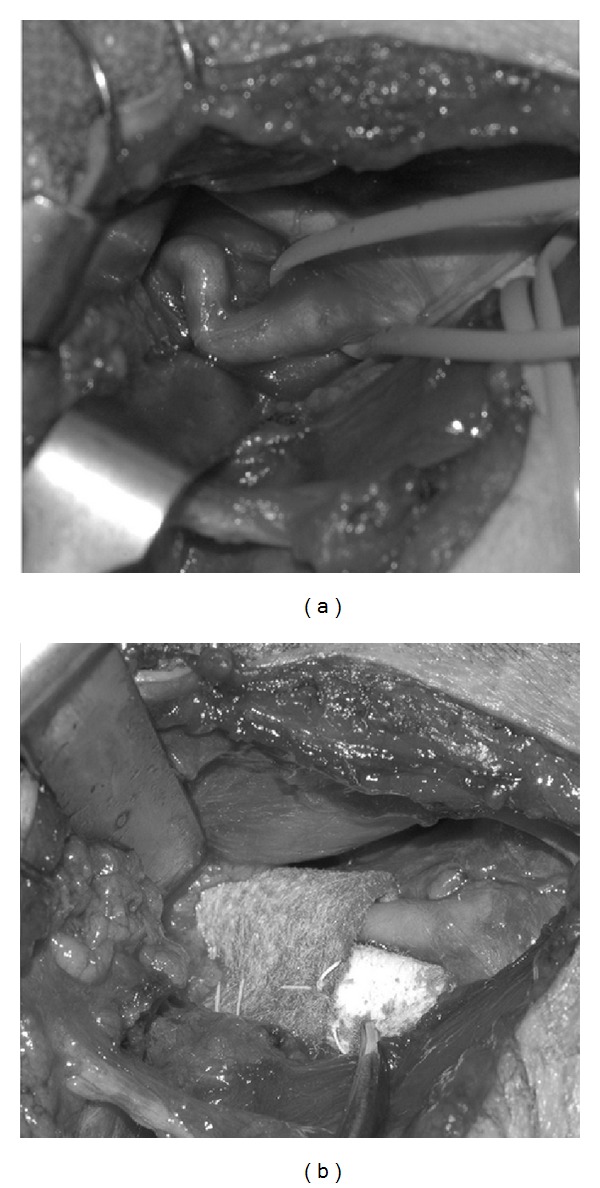
(a) Kink; (b) Teflon.

The patient was operated under general anesthesia. A right-sided semivertical incision was used to approach the common carotid artery. A segment of the common, external, and internal carotid artery was dissected from the surrounding tissue. A tortuous and kinked internal carotid artery was observed. The course of internal carotid artery was remodeled using a Teflon pledget and Gore-Tex sheet and 4-0 Gore-Tex sutures in such a way the severe kinking was corrected in a more straight course of the carotid artery (Figures 3(a) and 3(b)).

### 2.4. Postoperative Course

**Figure 4:**
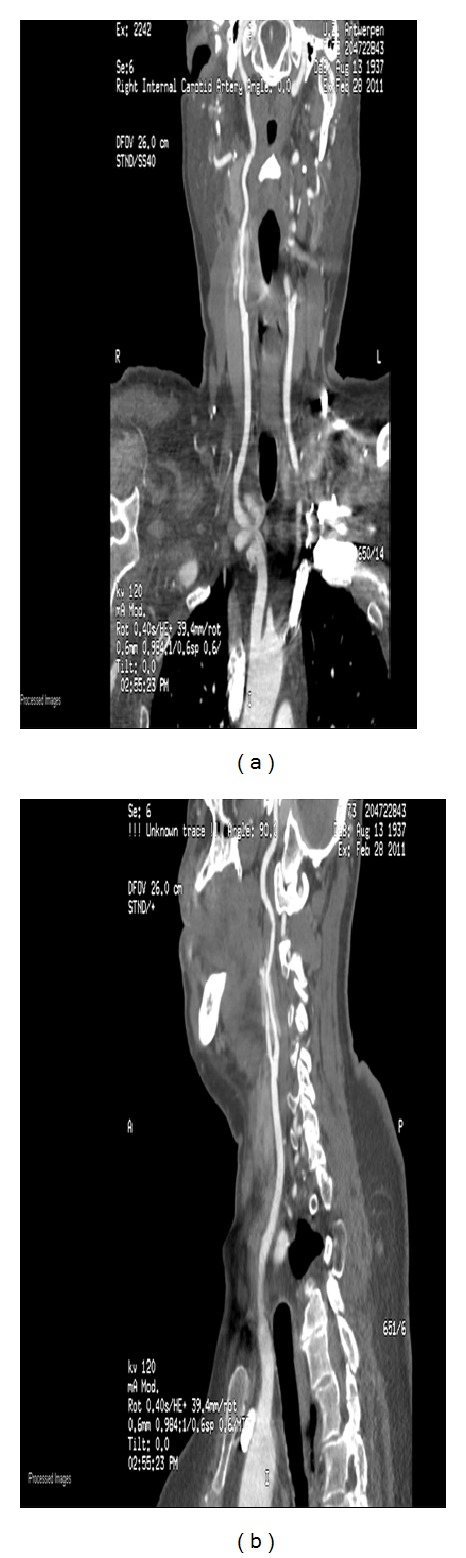
(a) AP view; (b) lateral view. The Teflon can be seen surrounding the ICA.

The postoperative course was uneventful. Immediately postoperatively, the patient experienced less pulsatile tinnitus than before the surgery. The patient was discharged on the third postoperative day. Postoperative CT-angiography performed six months after surgery showed a more straight course of the right extracranial internal carotid artery (Figure 4). 

The tinnitus is completely abolished during the night or in a reclined position, but not during the day. At a 1-year followup, the patient's tinnitus score was *5/10* on a visual analogue scale for loudness during the day, 0/10 during the night or when lying down. 

By adding a hearing aid, the tinnitus is almost completely abolished during the day (1/10), worsening to 2/10 in stressful situations.

## 3. Discussion

To the authors' knowledge, this is the first case operated by successful surgical remodeling of the internal carotid artery for pulsatile tinnitus. The positive arterial occlusion test demonstrated that the siphon-like abnormality of the internal carotid was causally related to the pulsatile tinnitus. It is likely that the turbulence caused by the siphon structure is generating the pulsatile tinnitus. 

There are multiple treatment options once the probable diagnosis is made by the carotid occlusion test. One is an end to end anastomosis after resection of part of the carotid, so that the artery is straightened and the turbulence arrested. This approach has as disadvantage that this intervention involves more risk, both at surgery and for inducing stenosis at the suture, resulting in pulsatile tinnitus, than the approach used by the authors, but the advantage that the pulsations might be completely abolished. 

Another option was stenting, using a long stent to straighten the siphon-like structure. But this has the risk of restenosing, resulting in pulsatile tinnitus and the risk of generating a siphon-like structure distally or proximally to the stent, also possibly resulting in recurrence of the pulsatile tinnitus.

After all these considerations, the option selected by the surgeons was to use an external straightening technique applying a soft material, Teflon, permitting an incomplete straightening but thereby preventing the reoccurrence of a siphon-like structure. The disadvantage of the selected technique is the incomplete success; that is, the tinnitus is only abolished during the night or when in a reclined position, but by adding a hearing aid, the tinnitus is nearly completely abolished, even during the day. After one year, the tinnitus improvement remains stable, scoring 1/10 during the day, with hearing aid, and 0/10 when reclined or going to sleep. The tinnitus score has improved to a grade 1, that is, a light form of tinnitus.

The reason the hearing aid further improved the tinnitus is likely linked to better masking of the arterial pulsations by external sounds. 

In summary, the results of this paper suggest that specialists treating patients with tinnitus should be aware of this vascular pathology as an underlying cause of pulsatile tinnitus, as it can lead to a successful treatment
